# Beyond PRP: Recognizing the Evolving Generations of Platelet Concentrates in Androgenetic Alopecia Treatment—A Letter in Response to Zhong Et al

**DOI:** 10.1111/jocd.71002

**Published:** 2026-06-24

**Authors:** Luo Ruiyu, Zhao Changjiang, Li Yufei

**Affiliations:** ^1^ Shanghai East Hospital, School of Medicine Tongji University Shanghai China; ^2^ School of Medicine Tongji University Shanghai China

To the Editor,

1

We read with great interest the comprehensive review by Zhong et al. regarding the application of platelet‐rich plasma (PRP) for androgenetic alopecia (AGA) [1]. The authors have provided a thorough synthesis of PRP preparation techniques, mechanisms of action, clinical efficacy, and safety, and have rightly emphasized the need for standardized protocols. We wish to extend this discussion by noting that platelet concentrate technology has evolved substantially beyond PRP over the past decade, and that a broader generational framework may offer a more complete perspective for clinicians and researchers.

Platelet concentrates for hair restoration are now widely recognized as spanning multiple generations. Building upon the first‐generation PRP, platelet‐rich fibrin (PRF) was introduced as a second‐generation product that eliminated the need for anticoagulants and provided a three‐dimensional fibrin scaffold for sustained growth factor release [2]. More recently, concentrated growth factor (CGF), a third‐generation preparation produced through variable‐speed centrifugation, has been shown to contain high concentrations of growth factors and preserve CD34+ progenitor cells within a denser fibrin matrix [3, 4].

The evolution did not stop at CGF. Further physical refinement has yielded a purified derivative—termed CGF‐purified fraction (CGF‐PF) or concentrated purified fraction (CPF)—that differs fundamentally from its predecessors. The purification process preferentially retains nanometer‐sized bioactive components, particularly extracellular vesicles and exosomes, while reducing larger particulate matter [5, 6]. Unlike the predominantly growth factor–based mechanism of PRP and PRF, CGF‐PF appears to mediate its effects through vesicle‐associated signaling. Studies have shown that exosomal microRNAs can significantly regulate hair follicle cycling and dermal papilla cell function [7], suggesting that the enhanced vesicle content of CGF‐PF may offer a distinct mechanism of action.

Preliminary clinical observations suggest that CGF‐PF offers improved injection tolerability and potentially more targeted modulation of hair follicle stem cell pathways. However, we acknowledge that prospective, controlled comparisons between CGF‐PF and conventional CGF are needed to establish its relative efficacy.

In summary, we commend Zhong et al. for their timely review of PRP therapy for AGA and suggest that the field may benefit from recognizing the full spectrum of platelet concentrate generations—PRP (first), PRF (second), CGF (third), and CGF‐PF/CPF (fourth)—as a useful reference framework. We look forward to future studies that may validate this classification through comparative clinical and mechanistic investigations.

## Funding

This work was supported by the Ministry of Education of the People's Republic of China, No. 231103242232720, No. 2408271251. Clinical Research Plan, No. SEHHH‐2021(KJ)‐0669‐KJB‐485, No. SEHHH‐2023(KJ)‐0143‐KYB‐111. Featured Clinical Discipline Project of Shanghai Pudong Fund, No. PWYts2021‐07. East Hospital Affiliated to Tongji University Introduced Talent Research Startup Fund, No. DFRC2019008.

## Conflicts of Interest

The authors declare no conflicts of interest.

## Linked Articles

This article is linked to Research Progress on Platelet‐Rich Plasma (PRP) in the Treatment of Androgenetic Alopecia, https://doi.org/10.1111/jocd.70809.

## Data Availability

The data that support the findings of this study are available from the corresponding author upon reasonable request.
